# Metabolomics Analysis of Soybean Hypocotyls in Response to *Phytophthora sojae* Infection

**DOI:** 10.3389/fpls.2018.01530

**Published:** 2018-10-23

**Authors:** Longming Zhu, Yang Zhou, Xiangnan Li, Jinming Zhao, Na Guo, Han Xing

**Affiliations:** National Center for Soybean Improvement, Key Laboratory of Biology and Genetics and Breeding for Soybean, Ministry of Agriculture, State Key Laboratory for Crop Genetics and Germplasm Enhancement, College of Agriculture, Nanjing Agricultural University, Nanjing, China

**Keywords:** soybean, defense response, metabolomics, *Phytophthora sojae*, resistant metabolites

## Abstract

Soybean is one of the most important economic and oil crops across the world. Phytophthora root rot (PRR), caused by *Phytophthora sojae* (*P. sojae*), is a major disease in most soybean-growing regions worldwide. Here, we investigated metabolic changes in hypocotyls of two soybean lines, Nannong 10-1 (resistant line, R) and 06-070583 (susceptible line, S), at two time points (12 and 36 hpi) after *P. sojae* infection and metabolic differences between the R line and the S line. In total, 90 differentially accumulated metabolites (DAMs) were identified after *P. sojae* infection; the levels of 50 metabolites differed between the R line and the S line. There are 28 DAMs that not only differentially accumulated between the R line and the S line but also differentially accumulated after *P. sojae* infection. Based on the changes of these DAMs in response to *P. sojae* infection in different lines and at different timepoints, and the differences in the contents of these DAMs between the R line and the S line, we speculated that DAMs, including sugars (monosaccharides and oligosaccharides), organic acids (oxalic acid, cumic acid), amino acid derivatives, and other secondary metabolites (mannitol, octanal, hypoxanthine, and daidzein etc.) may participate in the metabolic-level defense response of soybean to *P. sojae*. In this study, an integrated pathway-level analysis of transcriptomics (obtained by RNA-Seq) and metabolomics data illustrated the poor connections and interdependencies between the metabolic and transcriptional responses of soybean to *P. sojae* infection. This work emphasizes the value of metabolomic studies of plant–pathogen interactions and paves the way for future research of critical metabolic determinants of the soybean-*P. sojae* interaction.

## Introduction

Soybean (*Glycine max*) is one of the most important economic and oil crops worldwide. Phytophthora root rot (PRR), caused by *Phytophthora sojae* (*P. sojae*), is a major disease in most soybean-growing regions of the world (Tyler, 2007). *P. sojae* is a soil-borne oomycete pathogen that infects soybean plants at all developmental stages. Distinctive symptoms of *P. sojae* infection include damping-off, root and stem rot, and leaf yellowing and wilting, and the infection eventually results in seedling death; adult plants endure losses and complete yield reductions in susceptible soybean cultivars (Chang et al., 2017). This disease causes great yield loss every year and caused the loss of 0.68 × 10^6^ ∼ 1.55 × 10^6^ tons of yields from 1996 to 2009 in the United States soybean-growing regions alone (Wrather et al., 2001, 2003; Wrather and Koenning, 2006; Koenning and Wrather, 2010). The use of resistant soybean cultivars is the most economical, effective and environmentally friendly method of controlling this pathogen (Sahoo et al., 2017). Thus, the discovery of resistance resources and an improved understanding of the corresponding defense mechanisms are particularly important for the breeding of resistant cultivars.

‘Omics’ approaches, such as transcriptomics, proteomics and metabolomics, have been widely used in studies of plant–pathogen interactions to explore the plant defense mechanisms. Several studies have successfully characterized the interaction between soybean and *P. sojae* by ‘omics’ approaches. In particular, transcriptomics and proteomics were successfully applied to the studies of interaction between soybean and *P. sojae*. For example, a microarray study of transcriptomic changes in soybean revealed an upregulation of genes encoding enzymes of phytoalexin biosynthesis and defense and pathogenesis-related (PR) proteins (Moy et al., 2004). A microarray study of one susceptible and two partially resistant soybean genotypes infected with *P. sojae* showed that almost the entire plant genome (97–99% of all detectable genes) undergoes transcriptional modulation in response to infection and genetic variation (Zhou et al., 2009). The transcriptomes of 10 near-isogenic lines, each with a unique *Rps* (Resistance to *P. sojae*) gene/allele, and the susceptible parent Williams have been sequenced, analyzed, and compared. *Rps*-mediated defense response mechanisms are involved in ethylene (ET), jasmonic acid (JA), ROS (reactive oxygen species), and MAPK-signaling and also include WRKY transcription factors (Lin et al., 2014). Additionally, a proteomic study of changes in soybean lines resistant and susceptible to *P. sojae* identified 26 proteins that were significantly affected at various time points in the resistant soybean line and 20 proteins that were significantly affected in the sensitive soybean line (Zhang et al., 2011). These studies have improved our understanding of the interaction between soybean and *P. sojae*. Thus, approaches in comparative ‘omics’ are powerful tools to define defense response mechanisms.

Plants biosynthesize specialized metabolites to adapt to biotic stresses. A few examples of plant metabolites involved in biotic responses include compounds such as polyols, including mannitol, sorbitol, trehalose, fructan, proline, ectoine, the saponins, the glucosinolates, the phenolamides, the phenylpropanoids and the flavonoids (Shulaev et al., 2008). However, these characterized compounds are just the tip of the iceberg; more compounds that confer disease resistance have not been discovered due to the high diversity of metabolites in plant cells. Investigators have recently begun to harness the power of metabolomics with advances in analytical methods and data analysis to discover new disease resistance compounds (Tenenboim and Brotman, 2016). It is essential that metabolomics are combined with other “omics” approaches to obtain as comprehensive an overview as possible of cellular processes in a physiological context. In recent years, an increasing number of studies of plant abiotic/biotic interactions using integrated omics approaches have been reported (Sana et al., 2010; Masclaux-Daubresse et al., 2014; Sade et al., 2014; Tzin et al., 2015; Liu et al., 2016; Glaubitz et al., 2017). However, metabolomics has not been applied to studies of soybean-*P. sojae* interactions or *Rps*-mediated defense response mechanisms. Therefore, little is known about the defense response of soybean to *P. sojae* at the metabolic level.

In this study, we conducted a metabolite accumulation analysis of the resistant soybean line Nannong 10-1, which contains the *RpsJS* gene (Sun et al., 2014), and the susceptible soybean line 06-070583 in response to *P. sojae* infection using gas chromatography (GC)-mass spectrometry (MS) methods. We integrated the metabolomics data with transcriptomics data (obtained by RNA-Seq) using the KEGG pathway as a medium. Use of these approaches enabled us to gain molecular insights into metabolic level defense response mechanisms and to discover new disease resistance metabolites and their corresponding regulatory genes, thus providing more options and ideas for the future study of *RpsJS*-mediated defense response mechanisms as well as for breeding soybean for resistance.

## Materials and Methods

### Plant Materials and *P. sojae* Culture and Inoculation

The soybean lines Nannong 10-1 (containing *RpsJS* gene, Sun et al., 2014, R) and 06-070583 (susceptible, S) were obtained from the National Center for Soybean Improvement, Nanjing, Jiangsu province, China, and used for this study. Seeds of each soybean line were planted in sterilized vermiculite in plastic pots (diameter = 15 cm) and placed in the growth chamber at 25°C with a 14 h light/10 h dark cycle.

Each line of 8-day-old seedlings was separated into four groups, with each group containing at least 300 seedlings. Two groups were inoculated (IN) with *P. sojae* isolate JS08-12, which was grown on a V8 juice agar plates (10% Campbell’s V8 vegetable juice, 0.02% CaCO_3_, 1.5% Bacto-agar) for 5 days using the modified hypocotyl inoculation method (Sun et al., 2011), while the other two groups were mock-inoculated with V8 juice agar medium as control check (CK) samples. After inoculation, the seedlings were placed in a mist chamber (90% relative humidity) for 12 h and then transferred to a growth chamber at 25°C with a 14 h light/10 h dark cycle. Hypocotyls were sampled from inoculated and mock-inoculated seedlings by excising 2 to 3 cm across the wounded site at 12 and 36 h post-inoculation (hpi) and then placed immediately into liquid nitrogen before being stored at -70°C for subsequent experiments. Eight samples were prepared (S-12h-IN, S-12h-CK, S-36h-IN, S-36h-CK, R-12h-IN, R-12h-CK, R-36h-IN, R-36h-CK), and each sample contained at least 10 hypocotyls. A series of macroscopic observations marking the progress of disease was recorded on the inoculated hypocotyls (Supplementary Figure S1).

### GC-MS

Extraction and sample preparation were performed according to a protocol described previously (Farag et al., 2014). GC-MS was carried out on the Agilent 7890A-5975C GC-MS system (Agilent, United States). Each 1 μL aliquot of a derivative solution was subjected to analysis. Separation was carried out on a non-polar DB-5MS capillary column (30 m × 250 μm I.D., J&W Scientific, Folsom, CA, United States) with high purity helium as the carrier gas at a constant flow rate of 1.0 mL/min. The GC temperature programming began at 60°C, followed by 8°C/min oven temperature ramps to 125°C, 4°C/min to 210°C, 5°C/min to 270°C, and 10°C/min to 305°C, and a final 3 min maintenance at 305°C. The electron impact (EI) ion source was held at 260°C with a filament bias of -70 V. Full scan mode (m/z 50-600) was used, with an acquisition rate of 20 spectrum/second in the MS setting. To assess for biological variance, eight biological replicates for each sample were extracted, derivatized and analyzed in parallel under identical conditions. A quality control (QC) sample, prepared by mixing aliquots of all the samples, was analyzed using the same method as the analytic samples. The QCs were injected at regular intervals (every 16 samples) throughout the analytical run to provide a set of data from which repeatability could be assessed.

### Raw MS Data Analysis

ChromaTOF software (v 4.34, LECO, St. Joseph, MI, United States) was used to analysis the MS data from GC-MS. A CSV file was obtained with three-dimensional data sets that included sample information, retention time-m/z and peak intensities after alignment with a Statistic Compare component. Internal standards and any known pseudo positive peaks, including peaks generated by noise, column bleed and *N*,*O*-bis(trimethylsilyl)trifluoroacetamide (BSTFA) derivatization, were removed from the data set, and then the peaks from the same metabolite were combined. The clean data set was normalized using the sum intensity of the peaks in each sample.

### Multivariate Data Analysis

SIMCA-P software (v 14.0, Umetrics, Umeå, Sweden) was used for multivariate data analysis. Principal component analysis (PCA) and (orthogonal) partial least-squares-discriminant analysis [(O)PLS-DA] were carried out to visualize the metabolic alterations among experimental groups after mean centering and unit variance scaling. Variable importance in the projection (VIP) ranked the overall contribution of each variable to the (O)PLS-DA model, and those variables with VIP > 1.0 were considered relevant for group discrimination. In this study, the default 7-round cross-validation was applied with 1/7th of the samples being excluded from the mathematical model in each round, in order to guard against overfitting.

### Screening and Identification of Differential Accumulation Metabolite (DAM)

Both multivariate and univariate statistical significance (VIP > 1.0 and *P* < 0.05) were used as the thresholds for the significance of differences in metabolite accumulation. Metabolites were annotated with using several databases, including the NIST 11 standard mass spectral databases, Fiehn databases and reference standards available in our laboratory (which were linked to ChromaTOF software), on the basis of matching their mass spectra with those of authentic standards. A metabolite whose spectrum is 70% or more similar to the spectrum of a reference standard can be considered the same compound as that standard.

### Integration Analysis of Metabolomics Data and Transcriptomics Data

The integration analysis of metabolomics data and transcriptomics data (unpublished) was carried out on the basis of KEGG (Kyoto encyclopedia of gene and gnome^1^) pathway. Transcriptomics data were obtained by RNA-Seq, which were sequenced on the Illumina Hiseq platform, and the plant samples were taken simultaneously with the samples used by GC-MS. *P*-values ≤ 0.01 was used as the threshold to determine the significance in gene expression. Selected differentially expression genes (DEGs), which involved in DAMs metabolism were listed in Supplementary Excel S1.

## Results

### Overview of the Metabolomics Data of Soybean Hypocotyls in Response to *P. sojae*

**FIGURE 1:**
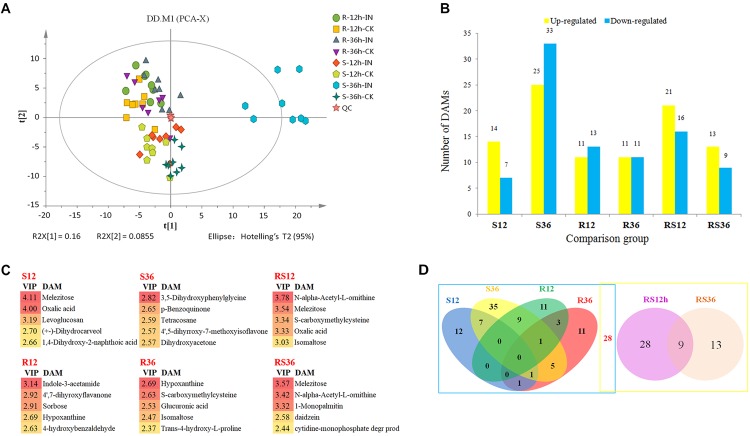
**(A)** Principal component analysis (PCA) of metabolites from soybean hypocotyls infected with *Phytophthora sojae* and those under control conditions. Each sample included eight biological replicates. **(B)** Numbers of significantly differentially accumulated metabolites (DAMs). DAMs are shown in yellow (upregulated) and blue (downregulated). **(C)** List of the DAMs with top 5 VIP value of each comparison group. **(D)** Venn diagram analysis of the DAMs. The left rectangle contains DAMs whose levels differ between infected hypocotyls and the corresponding control, and the right rectangle contains DAMs whose levels differ between the R and S lines. R12: R-12h-IN vs. R-12h-CK; R36: R-36h-IN vs. R-36h-CK; S12: S-12h-IN vs. S-12h-CK; S36: S-36h-IN vs. S-36h-CK; RS12: R-12h-CK vs. S-12h-CK; and RS36: R-36h-CK vs. S-36h-CK. IN, inoculated; CK, control check.

Metabolic profiling of the S line and the R line was carried out by GC-MS and detected 1,047 peaks in total. After the unvalued peaks were removed and the peaks from the same metabolite were combined, 311 metabolites were identified in samples using GC-MS (Supplementary Excel S2). To visualize the measurement reproducibility across the different biological replicates, the clean data that were normalized using the sum intensity of the peaks in each sample were subjected to PCA. The results showed that the biological replicates for each group were clustered in a small area (Figure 1A), suggesting high reproducibility. In particular, the reproducibility of QC was very high, suggesting that the whole experimental protocol and analysis methods were stable and reliable. All samples from the R line clustered in the upper left of the plot, while all samples from the S line clustered in the lower right of the plot, indicating that there were significant differences in the metabolite profiles of the R line and the S line. For each comparison group, the *P. sojae*-infected samples clustered to the left of their corresponding mock-infected samples, demonstrating that there was a change in the metabolic levels of soybean hypocotyls after *P. sojae* infection and that the magnitude of the changes depended on the extent of the disruption caused by *P. sojae* (Supplementary Figure S2). Six of the eight S-36h-IN samples clustered outside the confidence interval. Therefore, we rechecked the raw data and confirmed that it showed no abnormalities. Thus, the large differences between the metabolic profiles of the S-36h-IN samples and the other samples were due to the severe disruption caused by *P. sojae* and the presence of metabolites from *P. sojae* in the S-36h-IN samples.

All of the differentially accumulated metabolites (DAMs) were identified by comparing the metabolites of the *P. sojae-*inoculated groups and the corresponding control groups, as well as comparing the metabolites of the R and S lines. As a result, a total of 118 DAMs were identified (Supplementary Excel S3). Relative to the levels in mock-inoculated hypocotyls, the levels of 96 metabolites were significantly differentially accumulated in *P. sojae-*infected hypocotyls. Relative to the levels in the S line, the levels of 50 metabolites were significantly differentially accumulated in the R line across all timepoints (Figure 1B). The DAMs with top 5 VIP value of each comparison group are shown in Figure 1C. These compound, especially sugar such as melezitose, isomaltose, sorbose and secondary metabolites such as indole-3-acetamide, hypoxanthine, may play an important role during soybean response to *P. sojae* infection.

**FIGURE 2:**
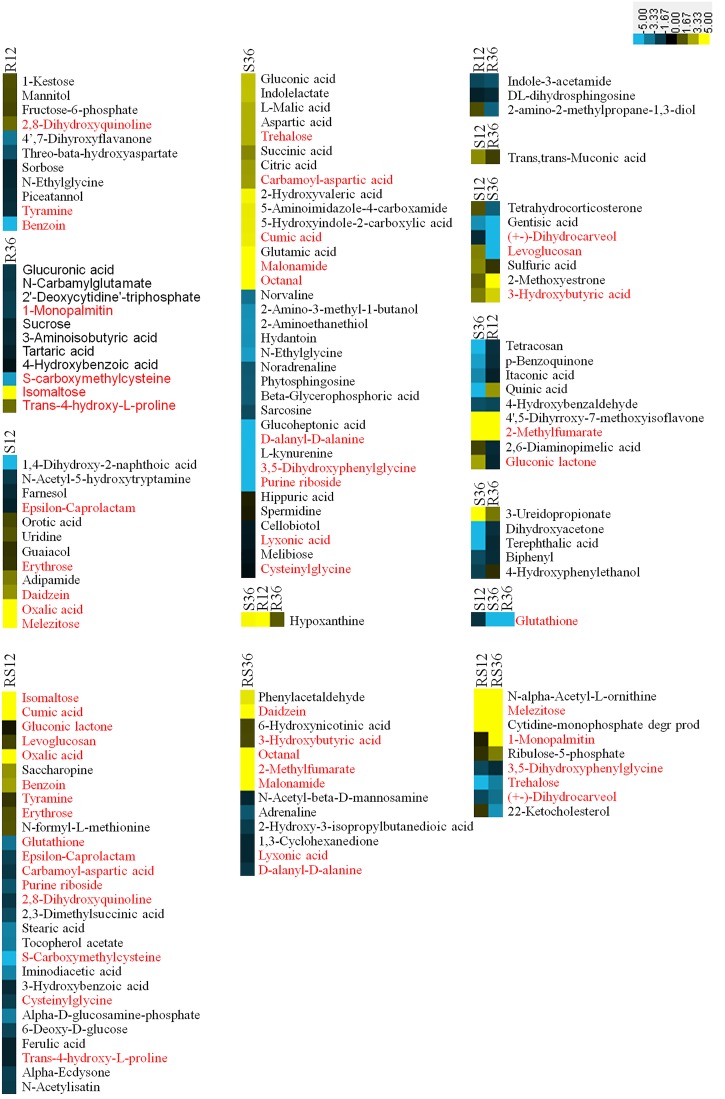
Heatmaps of DAMs from each subset of a Venn diagram. Metabolites in red font were differentially accumulated between the R line and the S line as well as being differentially accumulated after *P. sojae* infection. The log_2_Fold change was colored using Cluster 3.0 (yellow for upregulation and blue for downregulation), and each horizontal row represents a DAM with its name. R12: R-12h-IN vs. R-12h-CK; R36: R-36h-IN vs. R-36h-CK; S12: S-12h-IN vs. S-12h-CK; S36: S-36h-IN vs. S-36h-CK; RS12: R-12h-CK vs. S-12h-CK; and RS36: R-36h-CK vs. S-36h-CK. IN, inoculated; CK, control check. A color bar is in the upper right corner.

When these comparative results are illustrated as a Venn diagram, it is clear that both unique and shared DAMs occur between and among pairs (Figure 1D). For example, of the 96 DAMs that responded to the infection by *P. sojae*, 68 (69.4%) DAMs responded to the *P. sojae* infection at a specific timepoint, and 28 (30.6%) DAMs responded to the *P. sojae* infection at two or three timepoints. These results indicated that responses at the metabolic level are more time- and species-specific than responses at the transcriptional level. The combination of the Venn diagram and the heat map can help in speculating the role of each metabolite in the defense responses (Figure 2). For instance, 28 DAMs that not only differentially accumulated between the R line and the S line but also differentially accumulated after *P. sojae* infection (red font in Figure 2), as well as the DAMs identified in the R line (R12 and R36 subsets) after *P. sojae* infection, may have functions in resistance to *P. sojae.* In particular, the upregulated metabolites such as sugars (melezitose, levoglucosan, erythrose, trehalose, isomaltose, and 1-kestose), organic acids (oxalic acid, cumic acid), amino acid derivatives (tyramine, saccharopine, *N*-formyl-L-methionine, *N*-α-acetyl-L-ornithine, phenylacetaldehyde indole-3-acetamide, 4-hydroxybenzoic acid, *trans*-4-hydroxy-L-proline, treo-beta-hydroxyaspartate and *S*-carboxymethylcysteine) and other secondary metabolites (mannitol, octanal, hypoxanthine and daidzein) may play an important role in *RpsJS*-mediated defense response mechanisms. In contrast, DAMs that were identified uniquely in S12 and S36 (black font in S12 and S36 subsets of Figure 2) are more likely the by-products of stress that appears in cells because of the disruption of normal homeostasis by *P. sojae* infection than disease resistance-related metabolites.

### Changes in Carbohydrate Metabolism Upon *P. sojae* Infection

Among the DAMs, 21 metabolites were involved in carbohydrate metabolism pathways, such as starch and sucrose metabolism, glyoxylate and dicarboxylate metabolism, the TCA cycle, etc. (Supplementary Figure S3 and Supplementary Table S1). Several sugars, especially monosaccharides and oligosaccharides such as melezitose, levoglucosan, erythrose, trehalose and isomaltose, and organic acids such as oxalic acid and were induced to accumulate after *P. sojae* infection of the S line or the R line. Furthermore, the contents of 1-kestose and these metabolites, with the exception of trehalose, were higher in the R line than in the S line.

**FIGURE 3:**
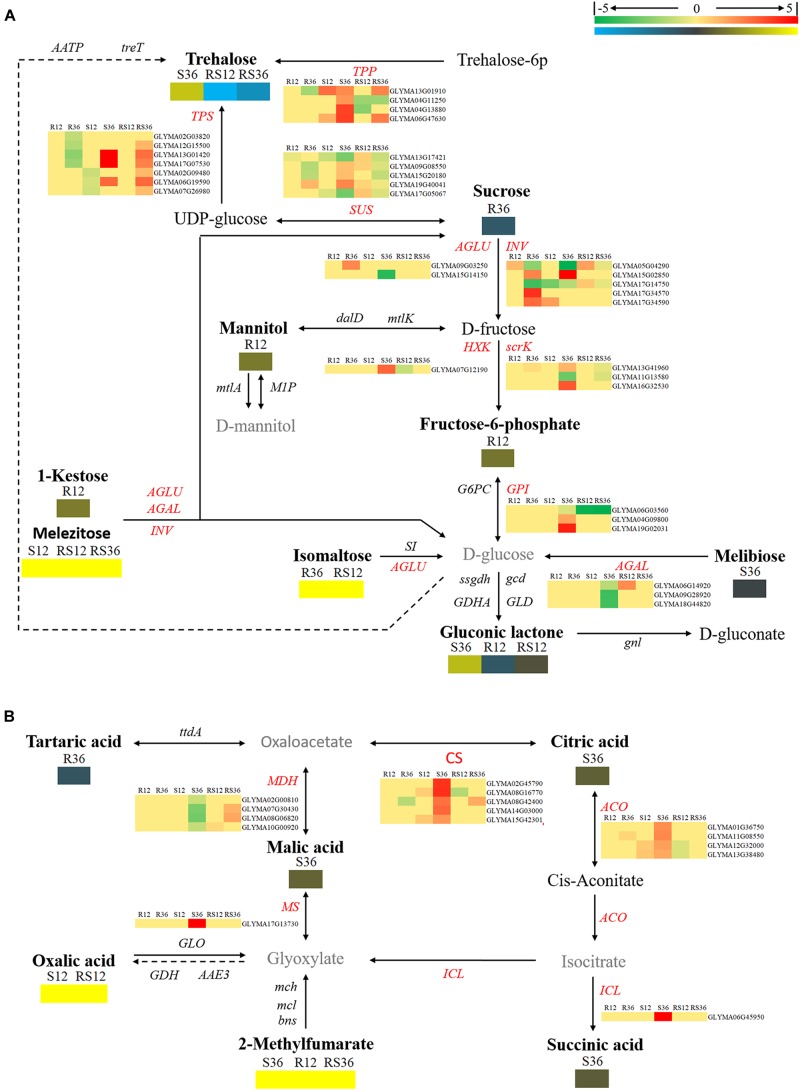
Diagram of carbohydrate metabolism pathways with their related DEGs (differentially expressed genes) and DAMs. **(A)** Pathway of starch and sucrose metabolism. **(B)** Pathways of the citrate cycle (TCA cycle), glyoxylate and dicarboxylate metabolism. DAMs are in bold black fonts. No significantly differentially accumulated metabolites are in black fonts; unidentified metabolites are in gray fonts. The abbreviations of gene names are in italics. The log_2_Foldchange was colored using Cluster 3.0 (red for upregulated DEGs, green for downregulated DEGs, yellow for upregulated DAMs, and blue for downregulated DAMs), and the vertical columns represent R12, R36, S12, S36, RS12 and RS36, from left to right. R12: R-12h-IN vs. R-12h-CK; R36: R-36h-IN vs. R-36h-CK; S12: S-12h-IN vs. S-12h-CK; S36: S-36h-IN vs. S-36h-CK; RS12: R-12h-CK vs. S-12h-CK; and RS36: R-36h-CK vs. S-36h-CK. IN, inoculated; CK, control check. A color bar is in the upper right corner. Abbreviations of gene names are listed in Supplementary Excel S4.

Based on the KEGG pathway, we also carried out an integrated analysis of the transcriptomics data and metabolomics data. In general, the oligosaccharides are converted by α-glucosidase (AGLU) into α-D-glucose and then fed into glycolysis. Nevertheless, several pieces of evidence and use of the schematic diagram showed that oligosaccharides such as 1-kestose, melezitose, trehalose, and isomaltose were not converted into glucose: first, the downstream product, α-D-glucose, was not differentially accumulated; second, transcripts (*Glyma09g03250* and *Glyma15g14150*) of AGLU were upregulated in R36 and downregulated in S36, respectively; and there was no significant difference in the expression levels between the R line and the S line. The expression of genes of TPP (trehalose 6-phosphate phosphatase, Glyma13g01910 and Glyma04g11250), that regulate trehalose synthesis, was changed in S36, R12 and R36, which consistent with the change of trehalose. The downregulated accumulation of sucrose in R36 may be due to upregulated expression of genes that encode sucrose-splitting enzymes such as *AGLU* (*Glyma09g03250*) and *INV* (*beta-fructofuranosidase*/*invertases, Glyma15g02850, Glyma17g34570, and Glyma17g34590*) in R36. While the change in the content of its downstream products (D-fructose) has not been identified (Figure 3A).

The levels of organics such as succinic acid, L-malic acid, and citric acid and genes encoding the enzymes citrate synthase (CS), malate dehydrogenase (MDH), isocitrate lyase (ICL), and aconitate hydratase (ACO), which are related to the TCA cycle, responded to *P. sojae* infection at 36 hpi in the S line. There is no change in expression of genes that regulate oxalic acid and 2-methylfumarate metabolism (Figure 3B).

### Changes in Amino Acid Metabolism Upon *P. sojae* Infection

**FIGURE 4:**
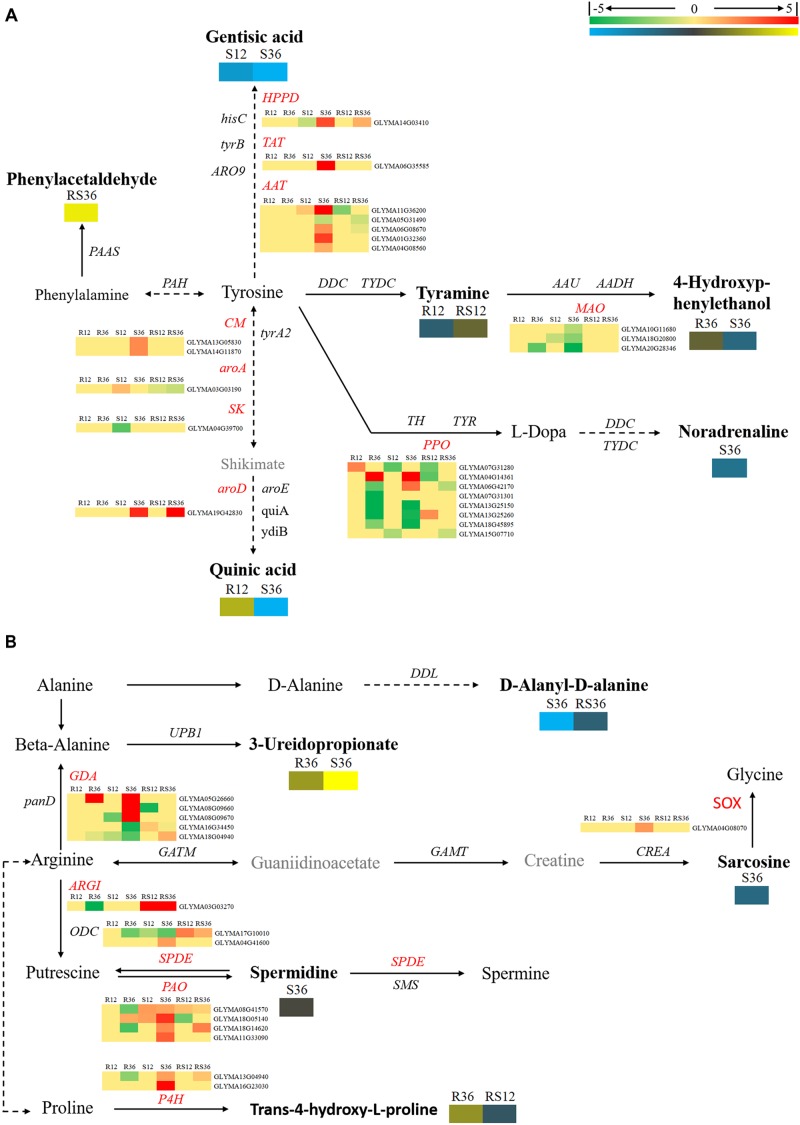
Diagram of amino acid metabolism pathways with their related DEGs and DAMs. **(A)** Pathway of tyrosine and phenylalanine metabolism. **(B)** Pathways of beta-alanine, proline, and arginine metabolism. DAMs are in bold black fonts. No significantly differentially accumulated metabolites are in black fonts, unidentified metabolites are in gray fonts, and the abbreviations of gene names are in italics. The log_2_Foldchange was colored using Cluster 3.0 (red for upregulated DEGs, green for downregulated DEGs, yellow for upregulated DAMs, and blue for downregulated DAMs), and the vertical columns represent R12, R36, S12, S36, RS12 and RS36, from left to right. R12: R-12h-IN vs. R-12h-CK; R36: R-36h-IN vs. R-36h-CK; S12: S-12h-IN vs. S-12h-CK; S36: S-36h-IN vs. S-36h-CK; RS12: R-12h-CK vs. S-12h-CK; and RS36: R-36h-CK vs. S-36h-CK. IN, inoculated; CK, control check. A color bar is in the upper right corner. Abbreviations of gene names are listed in Supplementary Excel S4.

**FIGURE 5:**
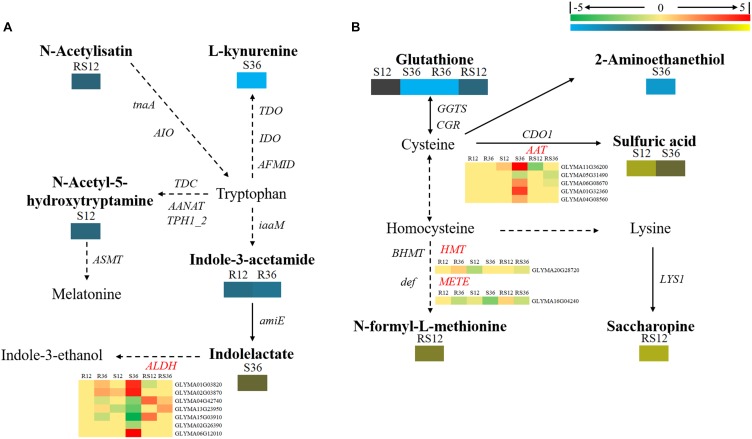
Brief diagram of amino acid metabolism pathways with related DEGs and DAMs. **(A)** Pathway of tryptophan metabolism. **(B)** Pathways of cysteine and lysine metabolism. DAMs are in bold black fonts. No significantly differentially accumulated metabolites are in black fonts, unidentified metabolites are in gray fonts, and the abbreviations of gene names are in italics. The log_2_Foldchange was colored using Cluster 3.0 (red for upregulated DEGs, green for downregulated DEGs, yellow for upregulated DAMs, and blue for downregulated DAMs), and the vertical columns represent R12, R36, S12, S36, RS12 and RS36, from left to right. R12: R-12h-IN vs. R-12h-CK; R36: R-36h-IN vs. R-36h-CK; S12: S-12h-IN vs. S-12h-CK; S36: S-36h-IN vs. S-36h-CK; RS12: R-12h-CK vs. S-12h-CK; and RS36: R-36h-CK vs. S-36h-CK. IN, inoculated; CK, control check. A color bar is in the upper right corner.

In this study, we identified a variety of amino acids and amino acid derivatives that were differentially accumulated after *P. sojae* infection and/or between the R line and the S line (Supplementary Figure S4 and Supplementary Table S2). Most of these metabolites differentially accumulated in response to *P. sojae* infection in the S line. In particular, all differentially accumulated amino acids such as sarcosine, glutamic acid, aspartic acid and norvaline were identified only in S36. The content of some amino acid derivatives, especially tyramine, saccharopine, *N*-formyl-L-methionine, *N*-α-acetyl-L-ornithine and phenylacetaldehyde, were significantly higher in the R line than in the S line. The levels of other metabolites such as indole-3-acetamide, tyramine, 4-hydroxybenzoic acid, *trans*-4-hydroxy-L-proline, treo-beta-hydroxyaspartate and *S*-carboxymethylcysteine responded to *P. sojae* infection in the R line. According to an integrated analysis of the transcriptomic data and metabolomic data, the correlation between the changes in metabolite contents and the changes in the expression of genes that encode the corresponding catalytic enzymes was not high (Figures 4, 5). Nevertheless, we determined that several genes, including *PAOs* (*polyamine oxidases*) and *SPDEs* (*spermidine synthases*), which are involved in the conversion between putrescine and spermidine; *ARGI* (*arginases*), which are involved in arginine metabolism (Figure 4); *ALDHs* (*aldehyde dehydrogenases*), which are involved in indolelactate downstream metabolism; *HMT* (homocysteine *S*-methyltransferase) and METE (5-methyltetrahydrofolate-homocysteine methyltransferase), which are involved in *N*-formyl-L-methionine upstream metabolism, may play important roles during the soybean response to *P. sojae* infection (Figure 5).

### Changes in Secondary Metabolism Upon *P. sojae* Infection

**FIGURE 6:**
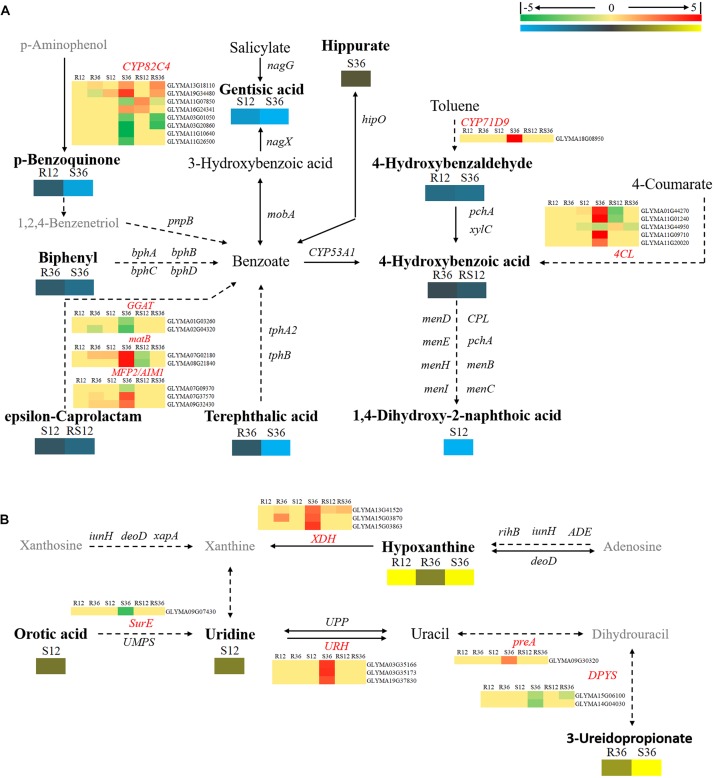
Diagram of secondary metabolism pathways with their related DEGs and DAMs. **(A)** Pathways centered on benzoate degradation. **(B)** Pathway of Purine metabolism. DAMs are in bold black fonts. No significantly differentially accumulated metabolites are in black fonts, unidentified metabolites are in gray fonts, and the abbreviations of gene names are in italics. The log_2_Foldchange was colored using Cluster 3.0 (red for upregulated DEGs, green for downregulated DEGs, yellow for upregulated DAMs, and blue for downregulated DAMs), and the vertical columns represent R12, R36, S12, S36, RS12 and RS36, from left to right. R12: R-12h-IN vs. R-12h-CK; R36: R-36h-IN vs. R-36h-CK; S12: S-12h-IN vs. S-12h-CK; S36: S-36h-IN vs. S-36h-CK; RS12: R-12h-CK vs. S-12h-CK; and RS36: R-36h-CK vs. S-36h-CK. IN, inoculated; CK, control check. A color bar is in the upper right corner. Abbreviations of gene names are listed in Supplementary Excel S4.

**FIGURE 7:**
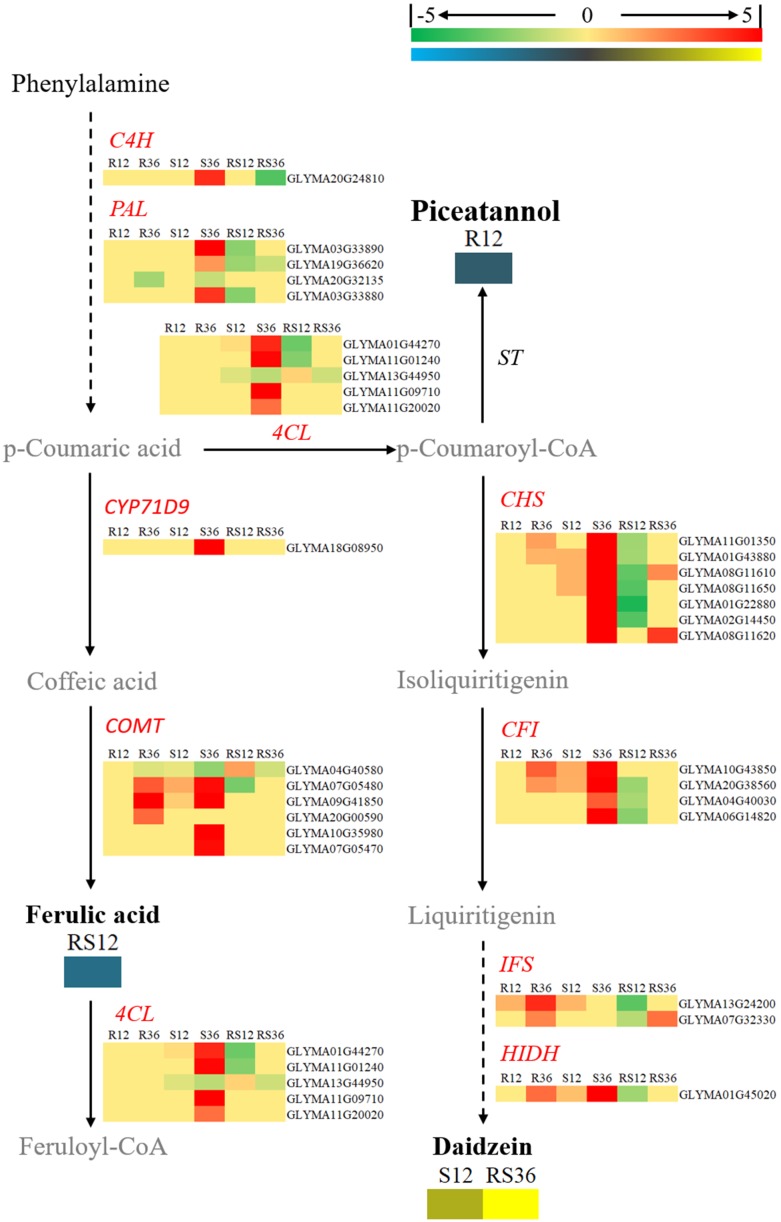
Diagram of a branch of the isoflavonoid biosynthesis pathway with its related DEGs and DAMs. DAMs are in bold black fonts. No significantly differentially accumulated metabolites are in black fonts, unidentified metabolites are in gray fonts, and the abbreviations of gene names are in italics. The log_2_Foldchange was colored using Cluster 3.0 (red for upregulated DEGs, green for downregulated DEGs, yellow for upregulated DAMs, blue for downregulated DAMs), and the vertical columns represent R12, R36, S12, S36, RS12 and RS36, from left to right. R12: R-12h-IN vs. R-12h-CK; R36: R-36h-IN vs. R-36h-CK; S12: S-12h-IN vs. S-12h-CK; S36: S-36h-IN vs. S-36h-CK; RS12: R-12h-CK vs. S-12h-CK; and RS36: R-36h-CK vs. S-36h-CK. IN, inoculated; CK, control check. A color bar is in the upper right corner. Abbreviations of gene names are listed in Supplementary Excel S4.

Changes in secondary metabolism are mainly concentrated in pathways centered on benzoate degradation (Figure 6A), purine metabolism (Figure 6B) and flavonoids, isoflavonoids biosynthesis (Figure 7). Most of the DAMs involved in benzoate degradation showed downregulated accumulation. Among them, p-benzoquinone, 4-hydroxybenzaldehyde, biphenyl, 4-hydroxybenzoic acid and terephthalic responded *P. sojae* infection in the R line. However, only *4CL* (*4-coumarate-CoA ligase*) and *CYP71D9* (*Cytochrome P450 71D9*), which are involved in 4-hydroxybenzoic acid and 4-hydroxybenzaldehyde upstream metabolism, respectively, showed differentially expressed in the S line (Figure 6A).

Among the DAMs that involved in purine metabolism, hypoxanthine and 3-ureidopropionate were upregulated accumulation in the R and S line. In particular, the VIP value of hypoxanthine is ranked in the top 5 in both R12 and R36. *XDHs* (*xanthine dehydrogenase/oxidase, Glyma13g41520* and *Glyma15g03870*), which involved in hypoxanthine downstream metabolism, were upregulated in R36 and S36. The remaining differentially expressed genes involved in purine metabolism only responded to *P. sojae* infection in S36 (Figure 6B).

Daidzein (4,7-dihydroxy isoflavone), a kind of isoflavonoid compound, was identified to accumulate in healthy R line plants but not in healthy S line plants; however, its content rapidly increased after infection with *P. sojae* in the S line. In terms of the key genes involved in the flavonoid and isoflavonoid biosynthesis pathways, *CHSs* (*chalcone synthases*), *CFIs* (*chalcone isomerases*), *IFSs* (*2-hydroxyisoflavanone synthases*) and *HIDH* (*2-hydroxyisoflavanone dehydratase*) were upregulated after *P. sojae* infection in both the R and S lines. In particular, the increase in the expression of *IFSs* and *HIDH* was more distinct. However, it appears that the key genes involved in the phenylpropanoid biosynthesis pathway such as *C4H* (*coumarate 4-hydroxylase*), *PALs* (*phenylalanine ammonia-lyases*) and *4CLs* (*4-coumarate-CoA ligases*) did not respond to *P. sojae* infection in the R line (Figure 7).

## Discussion

Metabolomic-based studies involving GC-MS analysis have great utility for exploring potential resistant metabolites and investigating metabolic response mechanism. This study identified several metabolites that may be resistant to *P. sojae*, including sugars (monosaccharides and oligosaccharides), organic acids, amino acid derivatives and other secondary metabolites. There are many reports on the importance of sugar levels in plant resistance to diseases caused by pathogens (Morkunas and Ratajczak, 2014). For example, trehalose (a non-reducing disaccharide), which showed differentially accumulated between the R line and S line an highly upregulated in S36, has been shown to partially induce resistance against powdery mildew (*Blumeria graminis* f. sp. *tritici*) in wheat by activation of phenylalanine ammonia-lyase and peroxidase genes (Reignault et al., 2001; Muchembled et al., 2006). In some cases, however, trehalose is essential for the infectivity of pathogens; specifically, in precise locations and at particular concentrations, trehalose can have a negative impact on plants (Fernandez et al., 2010). Thus, whether the high content of trehalose in the S line has a positive effect on soybean resistance to *P. sojae* is uncertain, which requires further study to explore. The 1-kestose (a non-reducing trisaccharide), which have a higher content in the R line than in the S line, may play a role in the defense response of *Arabidopsis* to nematode parasitism (Hofmann et al., 2010). Although there is no direct evidence that other oligosaccharides (melezitose, erythrose, isomaltose, and levoglucosan) that have a higher content in the R line than in the S line are involved in plant disease resistance, we still speculate that the high levels of these sugars may contribute to the high resistance of the R line to *P. sojae*. In addition to acting as carbon and energy sources (Koch, 2004), sugars, especially oligosaccharides, can also act as endogenous signal molecules to induce defense responses against pathogens (Rolland et al., 2006; Bolouri-Moghaddam et al., 2010; Bolouri-Moghaddam and Van den Ende, 2012; Morkunas and Ratajczak, 2014). Furthermore, oligosaccharides are generally involved in plant defense responses as substrates in plant cell wall (Shibuya and Minami, 2001). Thus, the high accumulation of oligosaccharides in the R line may contribute to signal transduction and plant cell wall modification during the response to *P. sojae* infection.

In addition to sugars mentioned above, other potential disease resistance metabolites identified in this study also have been shown by previous studies to be involved in biotic stress responses or resistance to pathogens. In particular, daidzein, the precursor of both coumestrol and the glyceollin phytoalexins, has been evidenced to play an important role in the defense response of soybean to *P. sojae* by silencing *GmIFS*, a key gene for the formation of isoflavonoids, including daidzein, in soybean cotyledon tissues, which decreased isoflavonoid content and enhanced susceptibility to *P. sojae* (Subramanian et al., 2005). Oxalic acid as a substrate to generate H_2_O_2_ under the catalysis of oxalate oxidase in plant, H_2_O_2_ can act as a strong oxidant against pathogens, and can also be used as a signaling molecule to induce disease-related gene expression, thereby enhance plant resistance to pathogens (Wu et al., 1995; Donaldson et al., 2001; Liang et al., 2001; Lane, 2002). Other studies indicated that exogenous oxalateinduced significant changes in relative abundance of a nunmber of *Brassica napus* proteins involved in stress reponse and redox homeostasis (Liang et al., 2009) and metabolic changes in sunflower (Monazzah et al., 2018). In addition, cumic acid and octanal have antifungal activity (Jayasinghe et al., 2003; Zhou et al., 2014).

Most of the potential disease resistance metabolites identified in this study, especially amino acid derivatives and hypoxanthine, have not been studied, and this study shows that these substances directly participate in the response to stresses. Some of these compounds are substrates for the biosynthesis of disease defense-related compounds. For instance, tyramine is the substrate for the biosynthesis of feruloyl-tyramine and coumaroyl-tyramine, which enhance the resistance of pepper to *Xanthomonas axonopodis* pv. *vesicatoria* and *X. campestris* pv. *campestris* (Newman et al., 2002). Much work must still be done to define the functions of these metabolites during responses to biotic stresses.

In this study, an integrated pathway-level analysis of transcriptomics and metabolomics data illustrated the poor connections and interdependencies between the metabolic and transcriptional responses of soybean to *P. sojae* infection. We speculate that there are three reasons for this behavior: first, there is a lag period between the metabolic and transcriptional responses to *P. sojae*, but we collected samples for analyses of both levels at the same times; second, the accumulation of metabolites and the expression of the corresponding regulatory genes have different degrees of change; for example, unmeasurable changes at the transcription level may cause measurable changes at the metabolic levels; third, changes in metabolite levels in plants in response to stress are not completely dependent on changes in transcription levels.

## Conclusion

This study provides new insight into the defense responses of soybean to *P. sojae* infection by examining metabolic changes in a time course. It seems that metabolites, including sugars (melezitose, levoglucosan, erythrose, trehalose, isomaltose, and 1-kestose), organic acids (oxalic acid, cumic acid and 2-methylfumarate), amino acid derivatives (tyramine, saccharopine, *N*-formyl-L-methionine, *N*-α-acetyl-L-ornithine, phenylacetaldehyde indole-3-acetamide, 4-hydroxybenzoic acid, *trans*-4-hydroxy-L-proline, treo-beta-hydroxyaspartate and *S*-carboxymethylcysteine) and secondary metabolites (daidzein, hypoxanthine, and octanal), may participate in the defense response. Although an integrated pathway-level analysis of transcriptomics and metabolomics data illustrated the poor connections and interdependencies between the metabolic and transcriptional responses of soybean to *P. sojae* infection, this study still identified some differentially expressed genes such as *INV, CHS, IFS, XDH* etc. were involved in the regulation of potential resistant substances. These findings in our experiments point the way for subsequent research projects such as studies of the *RpsJS*-mediated defense mechanism. Furthermore, future research on these findings will accelerate the breeding of soybean cultivars with enhanced resistance to *P. sojae* infection.

## Author Contributions

HX, NG, and JZ were the recipient of funds. LZ, NG, and HX conceived the experiment. LZ, YZ, and XL prepared the plant materials and collected samples. LZ, YZ, and JZ undertook experiments and data analysis. LZ prepared the manuscript.

## Conflict of Interest Statement

The authors declare that the research was conducted in the absence of any commercial or financial relationships that could be construed as a potential conflict of interest.

## Footnotes

1 http://www.genome.jp/kegg/
