# Supplementary material for: Metabolomics Analysis of Soybean Hypocotyls in Response to Phytophthora sojae Infection
Source: Front Plant Sci. 2018 Oct 23;9:1530. doi: 10.3389/fpls.2018.01530 (PMC6206292; doi:10.3389/fpls.2018.01530)
Supplement: Supplementary file 1 [file Data_Sheet_1.zip › supplementary figures.docx]

**Supplementary figures**

**
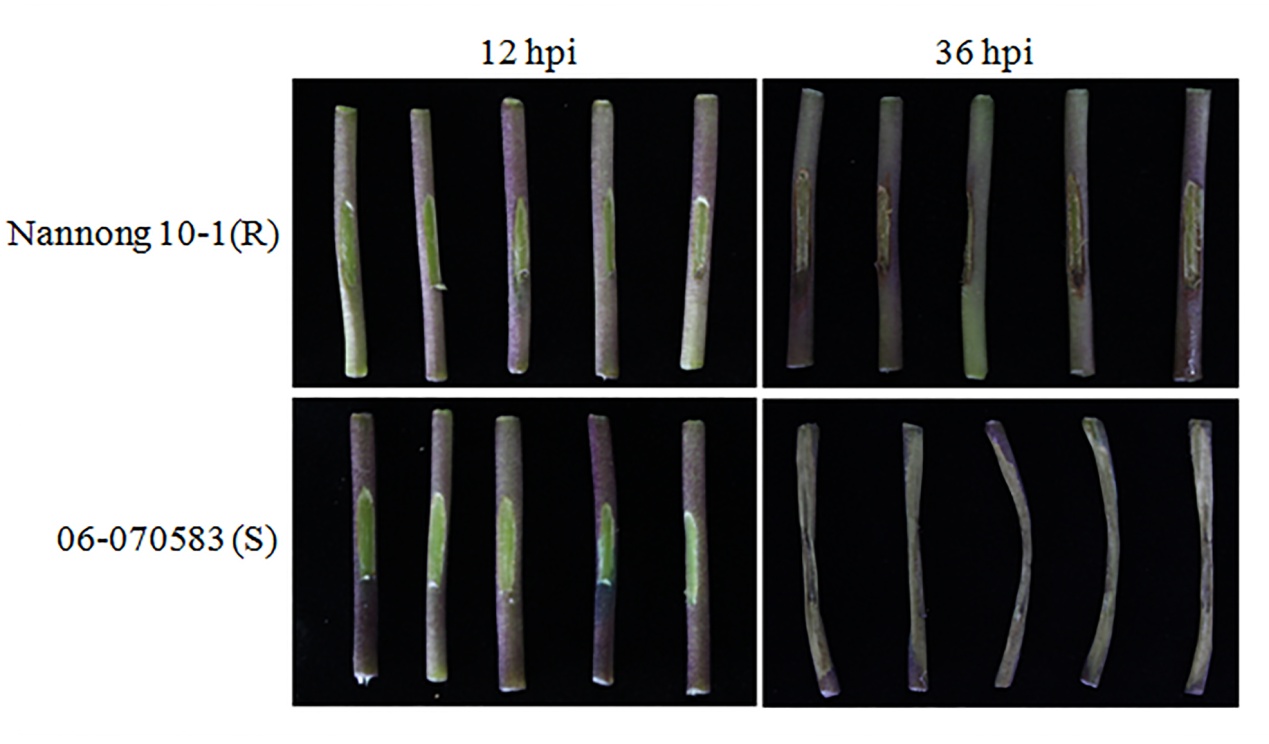
**

**Figure S1.** Changes in symptoms in soybean hypocotyls of Nannong10-1 and 06-070583 infection with ***Phytophthora*** *sojae* at 12 hpi and 36 hpi.


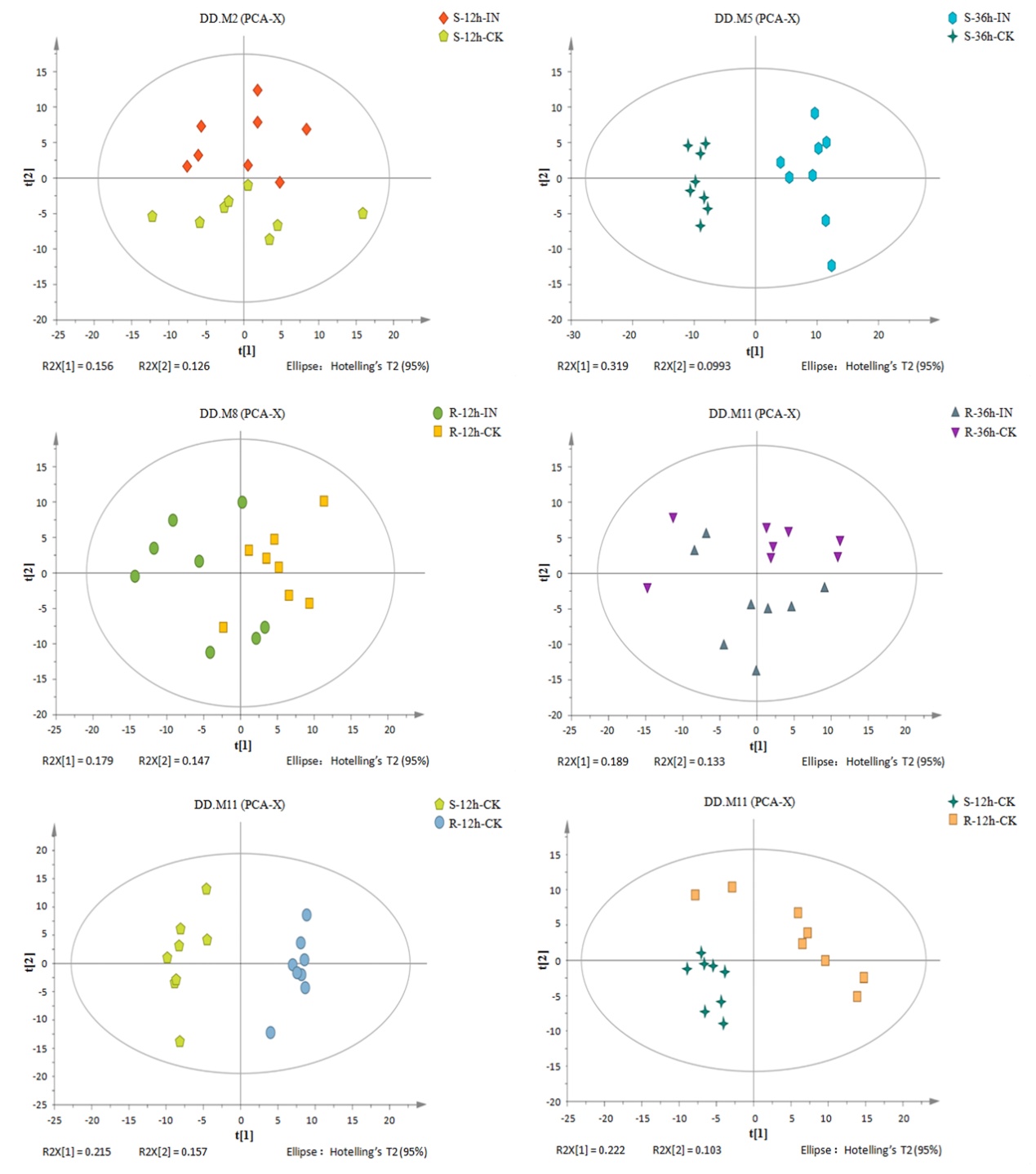


**Figure S2.** PCA analysis of each comparison group (S-12h-IN vs S-12h-CK; S-36h-IN vs S-36h-CK; R-12h-IN vs R-12h-CK; R-36h-IN vs R-36h-CK; R-12h-CK vs S-12h-CK; and R-36h-CK vs S-36h-CK).


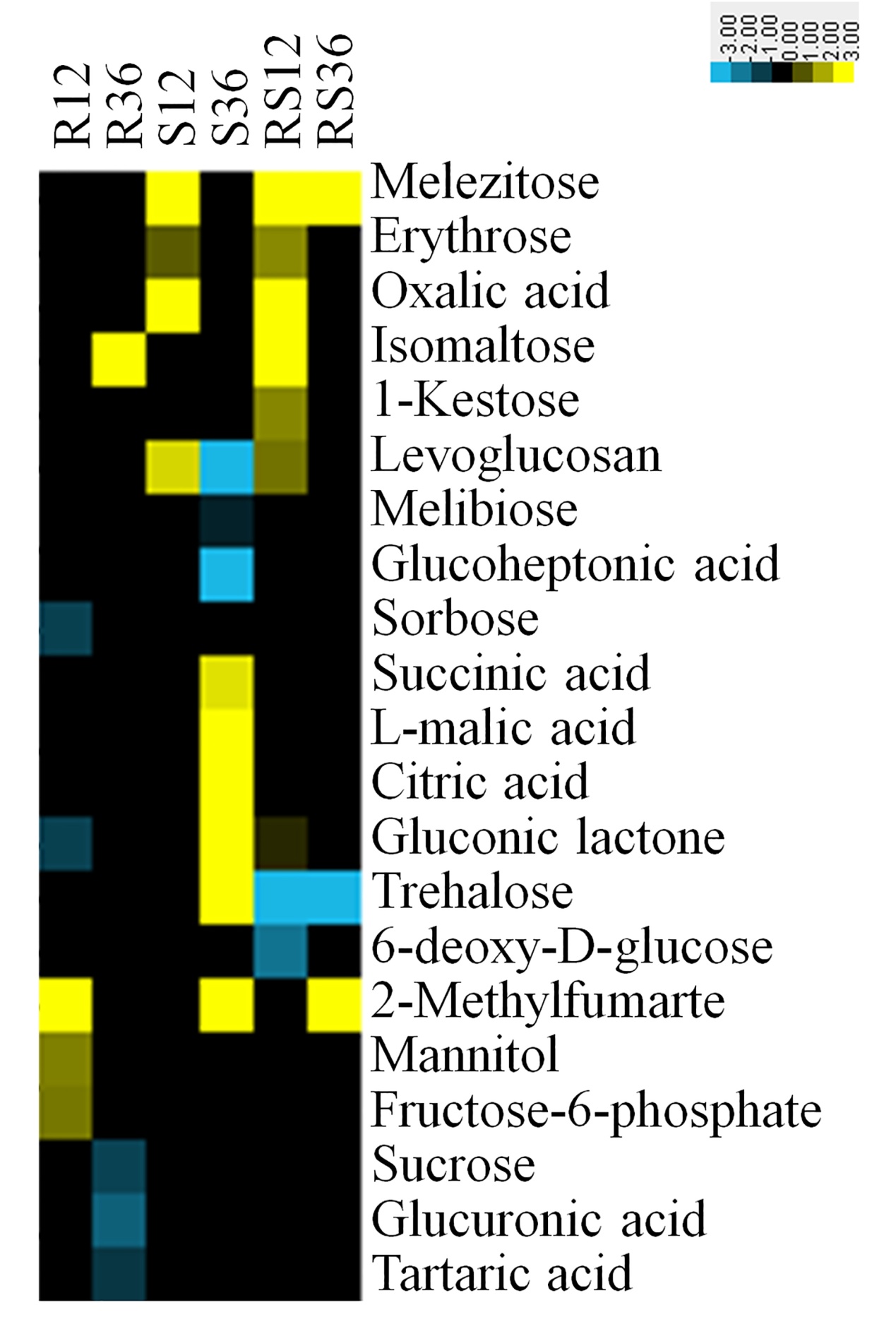


**Figure S3.** Heatmap of differentially accumulated metabolites (DAMs) related to carbohydrate metabolism pathways. The log_2_Foldchange was colored using Cluster 3.0 (yellow for up-regulated, blue for down-regulated), each horizontal row represents a DAM with its name, and the vertical columns represent R12, R36, S12, S36, RS12 and RS36 from left to right. R12: R-12h-IN vs R-12h-CK; R36: R-36h-IN vs R-36h-CK; S12: S-12h-IN vs S-12h-CK; S36: S-36h-IN vs S-36h-CK; RS12: R-12h-CK vs S-12h-CK; and RS36: R-36h-CK vs S-36h-CK. IN: inoculated, CK: control check.Color bar in the upper right corner.

**
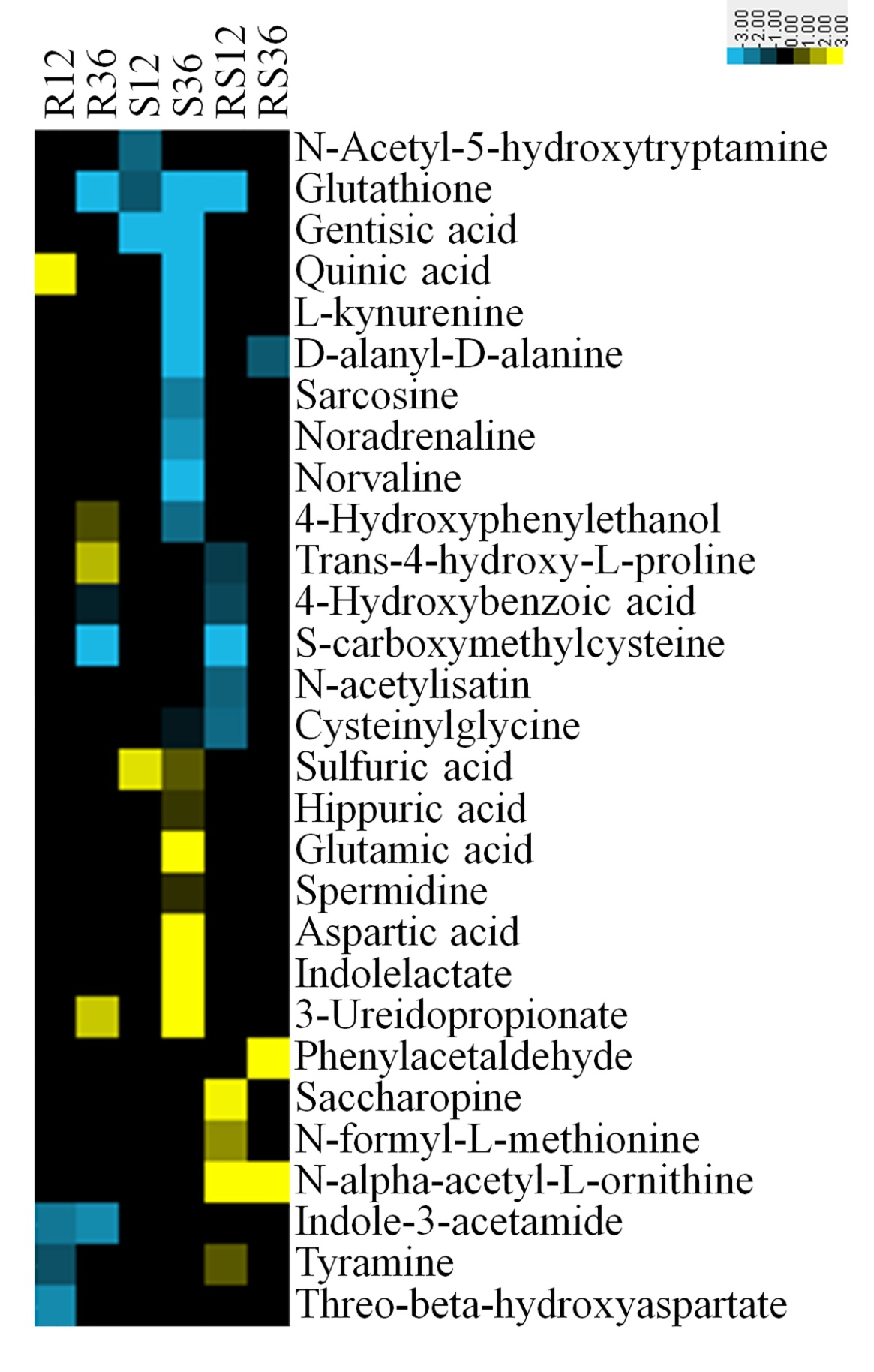
**

**Figure S4.** Heatmap of differentially accumulated metabolites (DAMs) related to amino acid metabolism pathways. The log_2_Foldchange was colored using Cluster 3.0 (yellow for up-regulated, blue for down-regulated), each horizontal row represents a DAM with its name, and the vertical columns represent R12, R36, S12, S36, RS12 and RS36 from left to right. R12: R-12h-IN vs R-12h-CK; R36: R-36h-IN vs R-36h-CK; S12: S-12h-IN vs S-12h-CK; S36: S-36h-IN vs S-36h-CK; RS12: R-12h-CK vs S-12h-CK; and RS36: R-36h-CK vs S-36h-CK. IN: inoculated, CK: control check.Color bar in the upper right corner.
