# Supplementary material for: Metabolomics Analysis of Soybean Hypocotyls in Response to Phytophthora sojae Infection
Source: Front Plant Sci. 2018 Oct 23;9:1530. doi: 10.3389/fpls.2018.01530 (PMC6206292; doi:10.3389/fpls.2018.01530)
Supplement: Supplementary file 1 [file Data_Sheet_1.zip › Supplementary Table 1. Metabolites related to carbohydrate metabolism pathways.docx]

**Supplementary Table 1. Metabolites related to carbohydrate metabolism pathways.**

| **Pathway** | **Significantly differentially accumulated metabolites** | **Nonsignificantly differentially accumulated metabolites** |
| --- | --- | --- |
| Galactose metabolism | Melibiose, Sucrose | N-Acetyl-D-galactosamine, Myo-inositol, Glycerol, Glucose-1-phosphate, Galactonic acid, Galactinol, Allo-inositol |
| Starch and sucrose metabolism | Sucrose, Trehalose, Isomaltose, Glucuronic acid, Melezitose | Trehalose-6-phosphate, Glucose-1-phosphate, D-galacturonic acid |
| Citrate cycle (TCA cycle) | Succinic acid, L-Malic acid, Citric acid | Pyruvic acid, Fumaric acid, Aconitic Acid |
| Pyruvate metabolism | Succinic acid, L-Malic acid | Pyruvic acid, Fumaric acid |
| Glyoxylate and dicarboxylate metabolism | Tartaric acid, Succinic acid, L-Malic acid, Oxalic acid, 2-methylfumarate, Citric acid | D-Glyceric acid, Beta-hydroxypyruvate, Aconitic Acid |
| Pentose phosphate pathway | Fructose-6-phosphate, Gluconic lactone | Pyruvic acid, Glucosaminic acid, Gluconic acid, D-Glyceric acid |
| Fructose and mannose metabolism | Mannitol | Beta-Mannosylglycerate |
