# Supplementary material for: Metabolomics Analysis of Soybean Hypocotyls in Response to Phytophthora sojae Infection
Source: Front Plant Sci. 2018 Oct 23;9:1530. doi: 10.3389/fpls.2018.01530 (PMC6206292; doi:10.3389/fpls.2018.01530)
Supplement: Supplementary file 1 [file Data_Sheet_1.zip › Supplementary Table 2. Metabolites related to amino acid metabolism pathways..docx]

**Supplementary Table 2. Metabolites related to amino acid metabolism pathways.**

| **Pathway** | **Significantly differentially accumulated metabolites** | **Nonsignificantly differentially accumulated metabolites** |
| --- | --- | --- |
| Cysteine and methionine metabolism | Glutathione, Sulfuric acid, N-Formyl-L-Methionine | Serine, Pyruvic acid, O-Succinylhomoserine, L-Homoserine, L-Cysteine |
| Tyrosine metabolism | Tyramine, Succinic acid, Gentisic acid, Noradrenaline, 4-Hydroxyphenylethanol | Tyrosine, Succinate semialdehyde, Pyruvic acid, L-Dopa,Fumaric acid, 4-Hydroxycinnamic acid |
| Tryptophan metabolism | N-Acetyl-5-Hydroxytryptamine, L-Kynurenine, Indolelactate, Indole-3-acetamide, N-Acetylisatin | Tryptophol, Melatonin, 5-Methoxytryptamine, 5-Methoxyindole-3-acetic acid |
| Glycine, serine and threonine metabolism | Sarcosine | Serine, Pyruvic acid, L-homoserine, L-cysteine, Glycocyamine, Glycine, D-Glyceric acid, Creatine degr, Beta-hydroxypyruvate |
| Arginine and proline metabolism | Sarcosine, Spermidine, Trans-4-Hydroxy-L-Proline | Urea, Spermine, Pyruvic acid, Putrescine, Glycocyamine, Glutamic acid, Creatine degr, 5-Aminovaleric acid, 4-Aminobutyric acid, 3-Hydroxy-L-proline, 1-Methylhydantoin |
| Phenylalanine, tyrosine and tryptophan biosynthesis | Quinic acid | Tyrosine, Shikimic acid, D-Fructose-1,6-Bisphosphate, 3-Hydroxybenzoic acid |
| beta-Alanine metabolism | Spermidine, 3-Ureidopropionate | Uracil, Spermine, Pantothenic acid, N-Acetyl-beta-alanine, Malonic acid, Beta-Alanine, 5,6-Dihydrouracil, 4-Aminobutyric acid, 3-Hydroxypropionic acid |
| D-Alanine metabolism | D-Alanyl-D-Alanine | Pyruvic acid |
| Phenylalanine metabolism | Hippuric acid, Phenylacetaldehyde, 4-Hydroxybenzoic acid | Vanillin, Tyrosine, Succinic acid, Salicylic acid, Pyruvic acid, Fumaric acid, Benzoic acid, 4-Hydroxycinnamic acid |
| Lysine biosynthesis | Saccharopine | Lysine, L-Homoserine, Alpha-Aminoadipic acid, 2,6-Diaminopimelic acid |
